# Avoiding Costly Conservation Mistakes: The Importance of Defining Actions and Costs in Spatial Priority Setting

**DOI:** 10.1371/journal.pone.0002586

**Published:** 2008-07-02

**Authors:** Josie Carwardine, Kerrie A. Wilson, Matt Watts, Andres Etter, Carissa J. Klein, Hugh P. Possingham

**Affiliations:** 1 The Ecology Centre, School of Integrative Biology, University of Queensland, Queensland, Australia; 2 CSIRO Sustainable Ecosystems, Queensland, Australia; 3 Department of Territorial Processes and Human Settlements, Faculty of Environmental and Rural Studies, Javeriana University, Bogotá, Colombia; University of Sheffield, United Kingdom

## Abstract

**Background:**

The typical mandate in conservation planning is to identify areas that represent biodiversity targets within the smallest possible area of land or sea, despite the fact that area may be a poor surrogate for the cost of many conservation actions. It is also common for priorities for conservation investment to be identified without regard to the particular conservation action that will be implemented. This demonstrates inadequate problem specification and may lead to inefficiency: the cost of alternative conservation actions can differ throughout a landscape, and may result in dissimilar conservation priorities.

**Methodology/Principal Findings:**

We investigate the importance of formulating conservation planning problems with objectives and cost data that relate to specific conservation actions. We identify priority areas in Australia for two alternative conservation actions: land acquisition and stewardship. Our analyses show that using the cost surrogate that most closely reflects the planned conservation action can cut the cost of achieving our biodiversity goals by half. We highlight spatial differences in relative priorities for land acquisition and stewardship in Australia, and provide a simple approach for determining which action should be undertaken where.

**Conclusions/Significance:**

Our study shows that a poorly posed conservation problem that fails to pre-specify the planned conservation action and incorporate cost *a priori* can lead to expensive mistakes. We can be more efficient in achieving conservation goals by clearly specifying our conservation objective and parameterising the problem with economic data that reflects this objective.

## Introduction

The global conservation community is charged with deciding where and how to invest limited funds to prevent biodiversity loss [1]–[4]. The conservation planning literature typically focuses on designing biological reserves [5]–[8] but in reality a variety of conservation actions are often under consideration to achieve conservation goals, e.g. land acquisition, invasive species control, and stewardship incentive payments to private land-holders [9]–[12]. Clear specification of conservation objectives and definition of conservation actions is thus essential. This involves parameterising our problems with relevant economic data, since the cost of each conservation action may vary differently throughout the land- or sea-scape [12], [13].

Spatially explicit cost data can be used in many available priority setting approaches, although examples are few. For example, systematic conservation planning is applied by Ando et al. [14] and Polasky et al. [15] to locate optimal places for land acquisition. Stewart and Possingham [16], Richardson et al. [17], and Klein et al. [18] design marine reserve networks that have minimal impact on fisheries. Balmford et al. [19] and Moore et al. [20] find cost-effective countries for managing land for conservation. Return on investments approaches have also been applied, typically on relatively coarse resolutions, to find optimal resource allocation strategies across regions [12], [21] or habitats [22]. Regardless of scale or resolution the message is consistent: large gains in efficiency can be achieved by explicitly accounting for the cost of conservation.

Economic considerations are commonly given far less attention in spatial conservation priority setting than biological values [3], [13]. Non-monetised surrogates such as the area of land or sea are typically employed as an implicit or explicit measure of conservation cost in each area [6], [23]–[25]. This approach makes the tacit and unlikely assumption that conservation actions cost the same everywhere. If cost is considered it is often included *post hoc*, typically in the implementation phase of planning to evaluate the cost of a plan [26], or alternative plans [27], [28]. Evaluating, or choosing amongst a range of sub-optimal solutions is another inefficient strategy.

One possible reason for the widespread neglect of the cost component in conservation priority setting may be the difficulty in obtaining adequate data. We acknowledge that most economic data, as per biological data, is fraught with uncertainty, but both are needed to make cost-effective conservation decisions [13]–[15]. We believe that a further important reason for the lack of consideration of economics in conservation planning is inadequate problem formulation: often areas are identified as ‘important’ with no clear statement of the overall objective of the prioritisation process, the conservation action required, or the cost of its implementation [11], [29]. The flawed perception prevails that biodiversity priority will be obfuscated or compromised unless based purely on biological information. In conservation planning problems targets amounts are typically inflexible and no compromise is made – meeting targets whilst minimizing the cost of our conservation actions simply makes efficient use of conservation funds.

Here we investigate the importance of clearly specifying conservation objectives and parameterising analyses with the cost of the specific conservation actions. We undertake a conservation planning assessment for Australia and consider two alternative conservation actions: land acquisition and stewardship. We use area, a spatially homogenous cost, as a baseline for comparison. Our objectives are to represent biodiversity targets for a range of biodiversity features (vegetation types, environmental domains, and distributions of birds and species of national environmental significance) whilst minimising (separately) area, acquisition costs, and the stewardship costs of selected candidate priority areas. We aim to (i) determine the financial efficiency gained by using the cost surrogate that most closely reflects the planned conservation action, (ii) examine the effect of different cost surrogates on the relative priority of candidate priority areas, and (iii) provide an approach for determining which conservation action is most cost-effective at any given location.

## Results

**Figure 1:**
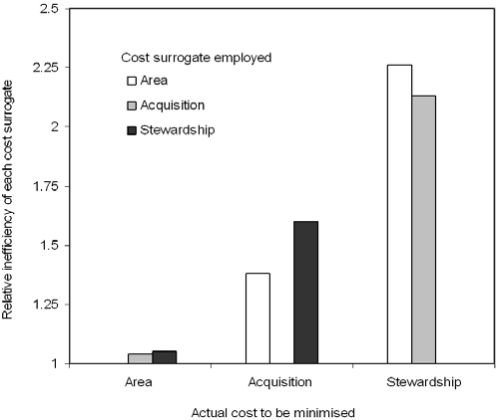
Relative inefficiency of using an inappropriate cost surrogate, compared to the minimum cost of meeting targets.

The most efficient solution for achieving each of our three objectives is obtained when using the cost surrogate that reflects the planned conservation action; we show a dramatic increase in the cost of achieving targets when the wrong cost surrogate is used (Figure 1). Inefficiency is shown relative to the most cost-efficient result, which is obtained by using the appropriate cost surrogate for the given conservation action. If our objective is to minimise stewardship or acquisition costs, but we had not specified our conservation objective and had used area is a cost surrogate, our targets would be 1.4–2.3 times more expensive to achieve. Conversely, solutions that minimised stewardship and acquisition resulting in a negligible area increase of 4–5%. Biodiversity targets would be more than twice as expensive to achieve by undertaking stewardship arrangements if acquisition cost is used as a surrogate. If stewardship cost is used but acquisition is undertaken, targets are only ∼0.6 times greater. The cost of acquisition and stewardship are not positively related at our study resolution (many of the areas that are costly to acquire are densely populated and have less value for agricultural production).

**Figure 2:**
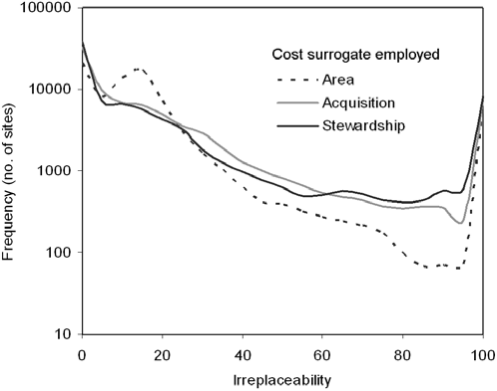
Frequency distribution of candidate priority area selection when using each cost surrogate.

Using spatially heterogeneous cost surrogates results in improved differentiation of relative priority, as measured by the selection frequency of each candidate priority area. A similar proportion of candidate priority areas are totally irreplaceable under each cost measure (Figure 2). However, the number of candidate priority areas selected >30% of the time is consistently higher when the cost measure employed is acquisition or stewardship, compared with area; and the number of candidate priority areas selected >80% of the time is substantially higher. When area is used a cost surrogate a greater number of areas are selected <30% of the time compared with when a spatially heterogeneous cost is used.

**Figure 3:**
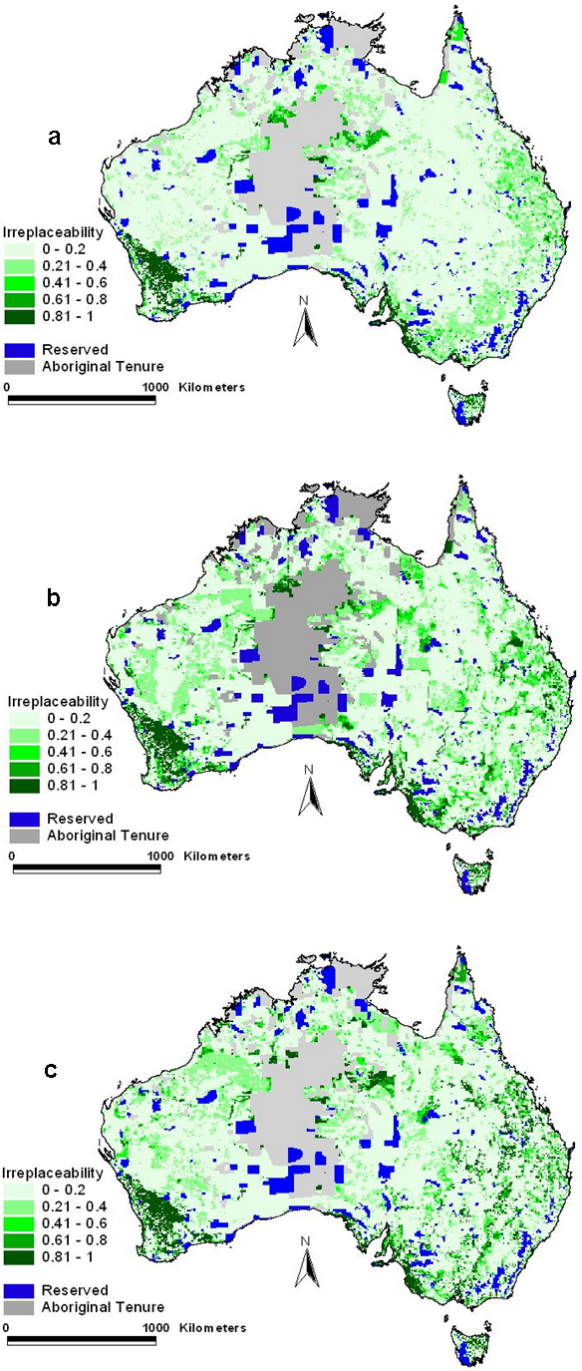
Relative priority of areas for each cost surrogate: a, area b, acquisition cost, and c, stewardship cost.

The subset of candidate priority areas that are irreplaceable (always selected) regardless of cost occur throughout the continent, e.g. in coastal areas and within the wheat belt of southern Western Australia where native vegetation clearing has left few options for meeting biodiversity targets (Figure 3). The relative priority for many candidate priority areas varies depending upon the cost measure employed. For example, some candidate priority areas in coastal regions are expensive to acquire, and are less favoured when the objective is to minimise acquisition compared with when the objective is to minimise area.

**Figure 4:**
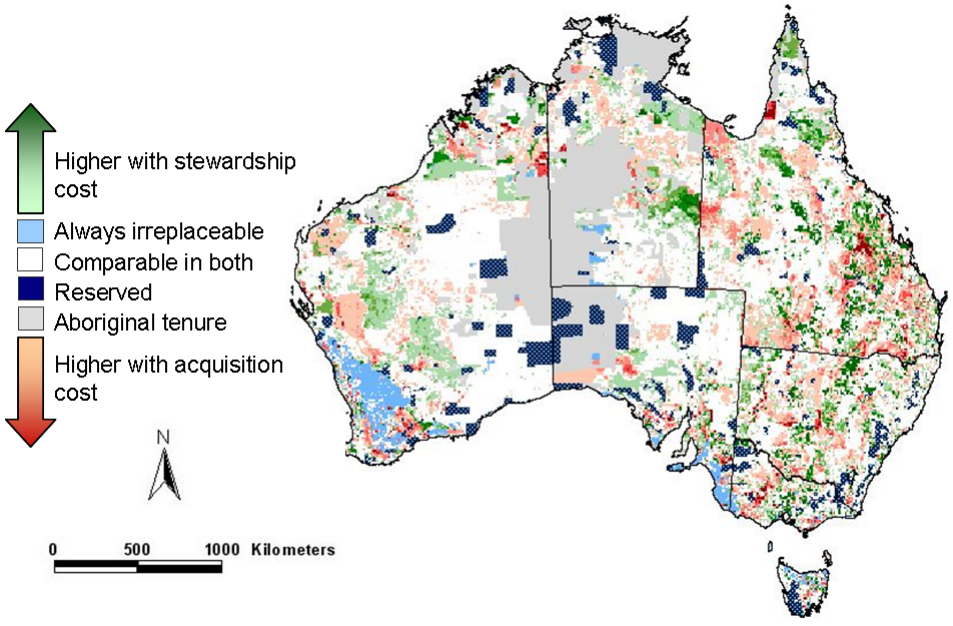
Differences in the relative priority of areas for two alternative conservation actions: acquisition and stewardship.

The information on relative priority under different scenarios can be combined to provide a map to indicate which action was allocated a higher priority in each location (Figure 4). Areas shaded blue are irreplaceable under either land acquisition or stewardship. Areas shaded green are likely to be cost-effective under stewardship arrangements compared to areas shaded red which are likely to be more preferable for acquisition. Candidate priority areas that are allocated substantially higher priority for acquisition are likely to offer more cost-effective options for land acquisition rather than stewardship, and vice versa. Differences in the relative values of these alternative cost surrogates result in some fine-resolution distinctions of which action is more favourable in a given candidate priority area.

## Discussion

We use a comprehensive set of ecological and economic data to identify cost-effective priority areas for undertaking land acquisition or stewardship arrangements across Australia. Our approach allows specific areas to be prioritised for investment in each alternative action. As per previous studies [14]–[18], we show that spatial misallocation of funds is likely to occur if conservation problems are not parameterised with actions and appropriate surrogates to estimate their cost. Our results also indicate that biodiversity targets can be up to twice as expensive to achieve by failing to correctly specify our conservation objective and parameterising analyses with relevant cost data.

We found that area is not a good surrogate for minimising the costs of land acquisition and stewardship. However, these spatially variable costs are effective at minimising area, because the same amount of area is required to meet our area-based conservation targets regardless of the cost surrogate employed see also [16]. Acting over a lesser total area of land (and number of properties) will help to minimise costs not accounted for in this study, such as the opportunity costs of some alternative land uses, and ongoing costs of management [13]. Area itself is a redundant surrogate for the cost of conservation in our study as it is minimised by spatially variable cost measures.

The difference in magnitude of the inefficiencies we observe between acquisition and stewardship cost is likely to be explained by the finer resolution of the stewardship layer: larger efficiency gains are therefore possible by accounting for it, but it is more difficult to minimise if it is ignored. The differences in resolution of the acquisition and stewardship datasets may also be responsible for some lack of correlation between these datasets – amortised annual profitability should be related to the cost of purchasing land in agricultural areas, assuming landowners are economic rationalists [30], although these values would diverge with distance from major centres and other social factors. There are shortcomings and inequalities in the two cost layers we employed. Our argument for increased consideration of costs in prioritisation assessments also highlights an urgent need for more accurate, current, and spatially-explicit economic data.

We observe an increase in the proportion of high priority areas when using a spatially variable cost surrogate. This indicates improved differentiation amongst among the areas selected to meet targets, but also some loss of flexibility: there are fewer cost-efficient options for representing our biodiversity surrogates than there are total (including inefficient) options see also [31]. This should help reconcile some site-based decisions. A subset of areas is totally irreplaceable regardless of the cost measure used. These high priority areas contain examples of rare biodiversity features for which there are few or no replacements in the landscape. Areas with lower priority do not necessarily have a low value for conservation – this simply indicates that there are more spatial options for investment to meet the targets for the features they contain [32]. Spatially explicit cost data can assist planners in representing biodiversity features cheaply where possible, while still ensuring that irreplaceable areas are allocated a high priority regardless of their cost.

Given that a combination of land acquisition for protected areas and stewardship arrangements on private land are often planned for [9], our approach can be used to determine which action to undertake in a given location. For example, our results suggest that acquisition is better targeted away from much of the comparatively costly land around cities, particularly on the eastern coast of Australia. Some areas within highly productive regions such as in the sheep and wheat belt may be less preferable for stewardship, as the compensation to landowners for forgone production is likely to be high. However, areas that are totally irreplaceable will be so regardless of the conservation action under consideration. These represent focal areas for conservation by either means, depending upon the local context and possibilities.

For practical application, our priorities may need to be refined by social and contextual aspects see [33], many of which can be difficult to address at a national scale. For example we have not considered management costs [19], threats [34], [35] and land-market feedbacks that may displace threats and affect costs [36]. In addition, a fine-resolution conservation planning approach that can synthesise multiple actions in a single scenario would provide a more definitive solution to which action is best to undertake in a given location. This is an important focus for further research, along with the generation of improved economic data. Therefore we demonstrate an approach that can be applied to other conservation problems, rather than a final set of priorities in Australia.

Decision making for conservation investments has so far escaped many of the requirements of standard investments, such as efficiency, goal setting and accountability [37], [38]. However, ignoring the cost of conservation actions is like shopping without price tags. Information on cost must be considered at some stage in the prioritisation process and we show the same biodiversity outcomes can be achieved with less expenditure if objectives are pre-specified and costs incorporated *a priori*. Concern amongst the global conservation community about limited funds and declining biodiversity has increased the demand for accountability [13], [39], [40]. It is essential for the credibility of future conservation planning analyses that we rise to this challenge.

## Materials and Methods

We divided the continent of Australia into candidate priority areas at a resolution of approximately 10*10 km^2^ (83,177 areas in total). Conservation actions involving the creation of habitat, such as restoration or revegetation were not considered in our analysis; hence we were only concerned with areas of remnant vegetation within these candidate priority areas. We used the freely available decision-support tool Marxan version 1.9.5 [7], which employs a simulated annealing algorithm to select multiple alternative sets of areas that meet pre-specified biodiversity targets (e.g. 3 populations of each species) whilst minimising a given cost, usually area. The proportion of times a site is selected estimates of the likelihood it is required to meet the given conservation objective, where irreplaceable sites are always required [41].

### Biodiversity features

We used four types of Australia-wide biological data, a total of 2590 biodiversity features, as biodiversity surrogates in our prioritisation: vegetation types, environmental domains, species distributions for non-vagrant birds, and distributions of floral and faunal species of national environmental significance. We masked out areas of each feature that occur in cleared areas. We applied targets of 15% of the pre-clearing extent for each biodiversity feature (except for birds where we use targets of 15% of current extent) for political reasons.

Vegetation types were represented by the Australian National Vegetation Information System (NVIS) 2001 dataset [42], with cleared areas masked out by [43]. The NVIS layer was intersected with the Interim Biogeographic Regionalisations for Australia [44], creating a more representative total of 1,763 vegetation types. Environmental domains were generated by Mackay et al. [45], and consist of 151 groups over the continent of Australia based on similarity of climatic, topographic, and substrate conditions (at a 250 m resolution). We also used point locality records from Birds Australia [46] (excluding ‘incidental’, pre 1985, and non-referenced sightings, and all introduced, vagrant, and wintering species, and sea birds, Hugh Possingham pers. comm), to generate models of the distribution of 563 bird species using alpha hulls, which are generalizations of convex hulls for creating surfaces from point data [47] to minimise the problems associated with false absences [48]. Finally we represented species of national environmental significance (those listed under the Australian *Environment Protection and Biodiversity Conservation Act 1999)*; we included modelled ranges of 1814 species classified as critically endangered, endangered, vulnerable, or conservation dependent [49].

### Cost surrogates

We used three alternative surrogates for the cost of conservation: area, acquisition and stewardship costs. In all cases we added a flat rate cost of 10,000 to each candidate priority area to represent a flat rate administrative or transaction cost required for undertaking any kind of conservation action.

First, we used the area of native vegetation in each candidate priority area (in ha) as a spatially homogenous cost.

Second, we estimated land acquisition costs using information on unimproved land value at a resolution of local government areas (n = 628, size: 9000 km^2^ (ave) 40000 km^2^, (max) 1 km^2^ (min), age: 1998–2005) sourced from State land valuation offices in Australia. We filled gaps in unincorporated lands and pastoral leases from the Northern Territory and Northern South Australia using (15*15 km^2^ grid) data on land sales [50]. We multiplied an area-weighted average unimproved value of each candidate priority area by the area of native vegetation in each, thus estimating the cost of acquiring the total area of native vegetation in each candidate priority area as per [51].

Third, we estimated stewardship costs, using data on average returns from agricultural production collected at 1 km resolution [52]. Profitability per hectare was estimated by subtracting the costs of production from gross revenue (yields×price), and averaged over 1992–97 to smooth annual fluctuations in consumer prices. We assumed land-owners are rational and would accept a once-off financial payment equal to their loss of potential production in perpetuity as per [30]. Negative profitability values, occurring where the overall cost of production outweighs the returns over much of Australia, were set to zero, to avoid under-estimating landholder values. We adjusted for inflation to the March quarter of 2006 [53] and multiplied the average profitability per hectare in each candidate priority area by its area of native vegetation. We determined the net present value (NPV) of forgone annual profitability in perpetuity over areas of native vegetation in each candidate priority area via:
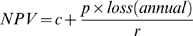
where:

c = flat rate administrative cost (set to $10,000)
r = discount rate (set to 6%)
p = portion of loss resulting from the stewardship arrangement (set to 50%)

The annual loss represents profits forgone if all production was forgone in areas of native vegetation within the candidate priority area. We assume that only a 50% reduction in productivity is required to undertake effective stewardship; however any other proportion or a variable proportion could be used.

### Scenarios and analysis

We ran Marxan 500 times using each of our three scenarios. Candidate priority areas that overlap by more than 50% of their area with existing protected areas (of IUCN status I–IV) were forced to be selected in every run. Land under aboriginal tenure is not obtainable for acquisition, so these areas were made unavailable for selection in all runs for consistency. We recognise that there is scope for valuable and cost-effective stewardship arrangements for biodiversity conservation on aboriginal land, and this is a focus of our ongoing research.

We used the best solution (that which meets targets at the minimum cost from 500 runs) in each scenario to compare the efficiency of each cost surrogate for achieving each of our three alternative objectives, i.e. to minimise the area, acquisition, and stewardship costs of representing targets. We used selection frequency as a measure of the relative priority of each area, although we recognise that other factors, e.g. threat could be used to refine this [41], [42]. We compared the frequency distribution in selection frequency and the spatial distribution in relative priority when using each cost surrogate. Finally, we determined the differences in relative priority for each candidate priority area under the alternative cost surrogates, and use this information to determine which action is more cost-effective in each area.
